# Barley yellow dwarf virus-GAV-derived vsiRNAs are involved in the production of wheat leaf yellowing symptoms by targeting chlorophyll synthase

**DOI:** 10.1186/s12985-020-01434-7

**Published:** 2020-10-21

**Authors:** Chuan Shen, Caiyan Wei, Jingyuan Li, Xudong Zhang, Qinrong Zhong, Yue Li, Bixin Bai, Yunfeng Wu

**Affiliations:** State Key Laboratory of Crop Stress Biology for Arid Areas and College of Plant Protection, Northwest A&F University, Yangling, 712100 China

**Keywords:** Barley yellow dwarf virus, Virus-derived siRNA, RNA silencing, Chlorophyll synthase

## Abstract

**Background:**

Wheat yellow dwarf virus disease is infected by barley yellow dwarf virus (BYDV), which causes leaf yellowing and dwarfing symptoms in wheat, thereby posing a serious threat to China's food production. The infection of plant viruses can produce large numbers of vsiRNAs, which can target host transcripts and cause symptom development. However, few studies have been conducted to explore the role played by vsiRNAs in the interaction between BYDV-GAV and host wheat plants.

**Methods:**

In this study, small RNA sequencing was conducted to profile vsiRNAs in BYDV-GAV-infected wheat plants. The putative targets of vsiRNAs were predicted by the bioinformatics software psRNATarget. RT-qPCR and VIGS were employed to identify the function of selected target transcripts. To confirm the interaction between vsiRNA and the target, 5′ RACE was performed to analyze the specific cleavage sites.

**Results:**

From the sequencing data, we obtained a total of 11,384 detected vsiRNAs. The length distribution of these vsiRNAs was mostly 21 and 22 nt, and an A/U bias was observed at the 5′ terminus. We also observed that the production region of vsiRNAs had no strand polarity. The vsiRNAs were predicted to target 23,719 wheat transcripts. GO and KEGG enrichment analysis demonstrated that these targets were mostly involved in cell components, catalytic activity and plant-pathogen interactions. The results of RT-qPCR analysis showed that most chloroplast-related genes were downregulated in BYDV-GAV-infected wheat plants. Silencing of a chlorophyll synthase gene caused leaf yellowing that was similar to the symptoms exhibited by BYDV-GAV-inoculated wheat plants. A vsiRNA from an overlapping region of BYDV-GAV MP and CP was observed to target chlorophyll synthase for gene silencing. Next, 5′ RACE validated that vsiRNA8856 could cleave the chlorophyll synthase transcript in a sequence-specific manner.

**Conclusions:**

This report is the first to demonstrate that BYDV-GAV-derived vsiRNAs can target wheat transcripts for symptom development, and the results of this study help to elucidate the molecular mechanisms underlying leaf yellowing after viral infection.

## Background

Barley yellow dwarf viruses (BYDVs) are a class of positive-sense single-stranded RNA viruses belonging to the family *Luteoviridae* [1]. These viruses were first discovered on barley in California in the United States in 1951 [2]. Subsequently, researchers observed that BYDVs can infect a variety of cereal crops, such as wheat (*Triticum aestivum*), barley (*Hordeum vulgare*), and oat (*Avena sativa*) [3]. BYDVs are phloem-limited and obligately transmitted by several cereal aphids in a persistent, circulative, and nonproliferative manner [4]. There are several distinct genera in this group, including BYDV-PAV, -PAS, -MAV, -Ker-II, and -Ker-III, which belong to the genus *Luteovirus*, CYDV-RPS, and CYDV-RPV, and -RMV, which belong to the genus *Polerovirus*, whereas BYDV-GPV and BYDV-SGV remain unassigned [5]. In addition, a similar serological reaction was observed between BYDV-GAV and BYDV-MAV. In recent years, BYDV-GAV has become the main pathogen of wheat yellow dwarf virus disease in China, which occurs widely and is prevalent in northwestern and northern China, causing serious economic losses [6, 7]. In the field, infection with BYDV-GAV can result in symptoms of leaf yellowing and plant dwarfism in several wheat cultivars [8].

**Fig. 1:**
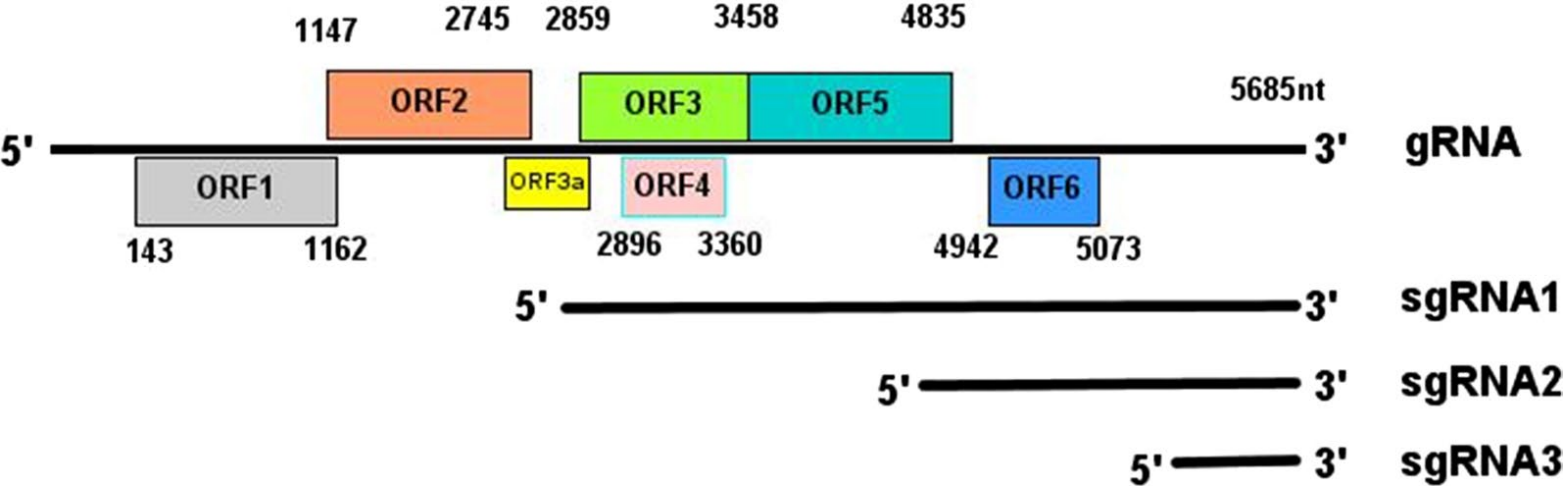
Genome of BYDV-GAV. ORF1: P1; ORF2: P2; ORF3: coat protein gene; ORF4: movement protein gene; ORF5: read-through domain; ORF6: P6; ORF3a: P3a

The genome of BYDV-GAV is approximately 5,685 bp and includes six open reading frames (ORFs) and four untranslated regions (UTRs) with no 5′ cap and 3′ poly(A) tail. ORF1 and ORF2 encode the P1 protein and the P1-P2 fusion protein, respectively, which are involved in viral genome replication. ORF3 encodes the coat protein (CP), ORF4 encodes the movement protein (MP), ORF5 encodes the CP read-through domain (RTD) in the 3′ terminus, and ORF6 encodes the P6 protein (Fig. 1) [7].

The plant RNA silencing mechanism is a natural and highly conserved molecular antiviral defense system utilized by plants against invasive viruses, whereas viruses could encode silencing suppressors to restrict host RNA silencing [9, 10]. Viral infection accompanied by the replication of RNA viral genomes by the host's RNA-dependent RNA polymerase (RDRP) and induced the production of double-stranded RNA (dsRNA) or the secondary structure of the single-stranded viral RNA, which are subsequently cleaved by the host plant Dicer-like (DCL) enzymes, which represent a major component of RNAi machinery, into small interfering RNAs (vsiRNAs) [11, 12]. These vsiRNAs are loaded onto AGO (Argonaute) family proteins and form the RNA-induced silencing complex (RISC) to perform posttranscriptional gene silencing, which could target the complementary sequences of the viral genome for specific degradation [13–15]. Previous studies have shown that the 21-nt vsiRNAs produced by a DCL4 enzyme guide the degradation or translational inhibition of transcripts targeted by the RISC, while the 24-nt-long vsiRNAs are often recruited to AGO4 proteins and induce systemic silencing and RNA-directed DNA methylation (RdDM) [16–18].

The secondary siRNAs generated from dsRNA can strengthen the function of the primary siRNAs, thereby promoting the systemic spread of silencing signals continuously [19]. Similarly, vsiRNAs might have a potential inhibition and targeting effect on host transcripts, which determines the development of viral symptoms in the host plant [20, 21]. These studies indicated that vsiRNAs not only target viral RNA but also complementary-sequence host transcripts, and the host disease symptoms are primarily due to vsiRNA-directed silencing of the targeted host genes [22, 23].

The interactions between host mRNAs and vsiRNAs and the cleavage of host mRNAs by vsiRNA have been studied widely. For example, by small RNA sequencing and 5′ RACE analysis, 22-nt siRNAs derived from cucumber mosaic virus (CMV) Y-satellite RNA (Y-Sat) were determined to target the cleavage of the chlorophyll synthesis gene (CHLG), which induces yellowing symptoms in the leaves of infected *N. benthamiana* (*Nicotiana tabacum*) [24]. VsiRNAs derived from the rice stripe virus (RSV) RNA4 were complementary to part of the host transcription initiation factor 4A (eIF4A) mRNA, resulting in the downregulation of target gene expression levels and inducing the symptoms of chlorosis, leaf twisting and stunting of *N. benthamiana* [20]. VsiRNAs derived from a rice virus had the potential to induce abnormal phenotypes by targeting rice endogenous transcripts in rice [25]. Two peach latent mosaic viroid (PLMVd)-derived siRNAs specifically target cleavage of the mRNA encoding chloroplastic heat-shock protein 90 (cHSP90), resulting in the albinism of peach leaves [26]. A virus-derived small interfering RNA generated from Chinese wheat mosaic virus (CWMV) RNA1 can target the transcripts of *TaVP* in wheat [27].

Virus infection often causes host plants to show symptoms of leaf yellowing. This phenomenon is usually associated with the impairment of chlorophyll biosynthesis, which leads to a decrease in photosynthesis [28, 29]. Several previous studies have reported that viral infection causes a reduction in chloroplast-related genes (ChRGs) or photosynthesis in plants. For example, turnip mosaic virus (TuMV) infection reduced the efficiency of photosynthesis in stem mustard [30]. Sugarcane yellow leaf virus (ScYLV) infection caused a reduction in photosynthetic efficiency and carbohydrate accumulation in sugarcane leaves [31]. Rice stripe virus (RSV) was observed to interact with PsbP, leading to an increase in disease symptom severity and virus accumulation in both rice and *N. benthamiana* cells [32]. Sugarcane mosaic virus (SCMV) infection resulted in a significant decrease in ferredoxin-5 (Fd V) mRNA levels in maize and disrupted the import of Fd V into chloroplasts [33]. The chlorosis produced by tobacco mosaic virus (TMV) infection was primarily due to the reduction of specific proteins of the photosystem II (PSII) core complexes and the 33-kDa protein of the oxygen-evolving complex [34]. In addition, the development of leaf yellowing symptoms in African cassava mosaic virus (ACMV) infection might be related to chlorophyll degradation and decreased expression levels of genes encoding light-harvesting complex II [35]. The chloroplast- and photosynthesis-related genes (CPRGs) were downregulated in the chlorotic tissues of tobacco plants after CMV inoculation, suggesting that the severity of chlorosis was correlated with the downregulation of CPRGs [36]. The results in the transcriptome of RSV-infected rice plants showed that RSV infection suppressed the expression of chloroplast-related genes, suggesting that the chlorosis symptom in the leaves of RSV-infected plants may be related to the decreased level of ChRGs [37].

Previous studies indicated that BYDV-GAV infection resulted in a decrease in chlorophyll accumulation and starch synthesis, which contributed to the development of leaf yellowing symptoms [38]. Chlorophyll synthase functions in the last step of the chlorophyll biosynthetic pathway, which plays an important role in the coordination of the synthesis of chlorophyll and chlorophyll-binding proteins [39]. With the widespread application of small RNA sequencing, research on the identification of the origin of vsiRNA and its function on host target genes has also advanced considerably, revealing that vsiRNAs have the potential to regulate gene expression. Our study found that vsiRNA8856 could target wheat CHLG transcripts to regulate their expression and induce the production of leaf yellowing symptoms.

In our study, we performed deep sequencing of small RNAs to analyze the vsiRNA profiles of BYDV-GAV-infected wheat and to identify their bioinformatic characteristics and derivation from the genome of the virus. We also predicted and validated the target of the vsiRNAs in wheat and their effects on wheat gene expression levels, thereby exploring the role of vsiRNAs as pathogenicity determinants of BYDV-GAV by targeting host transcripts. Our results help to elucidate the molecular mechanism governing the effects of vsiRNAs on the development of BYDV-GAV disease symptom and the molecular interactions between the virus and its host plant.

## Methods

### Plant growth and virus inoculation

The Xiaoyan 6 wheat plants were grown in a growth chamber with a cycle of 16 h light (24 °C) and 8 h dark (18 °C); the relative humidity was 65%. The 3- to 4-leaf-stage plants were inoculated by aphids that were fed on BYDV-GAV-infected *Avena sativa* cv. coast black plants for 3 d to acquire the virus. The aphids were killed by pesticides 3 d later. Wheat leaves were inoculated with viruliferous aphids carrying BYDV-GAV, and control leaves were inoculated with nonviruliferous aphids; five aphids per leaf were employed.

### Total RNA extraction and small RNA sequencing

Samples were harvested from wheat leaves at 7 d after infection with BYDV-GAV. Total RNA was extracted from each sample following the protocol described by Furtado (2014), employing a TRIzol kit (Invitrogen) and a Qiagen RNeasy Plant Mini Kit (Qiagen), with three independent biological replicates. Next, all these samples were immediately frozen in liquid nitrogen for over 10 min and stored at − 80 °C. The concentration of each RNA sample was measured using a QubitFluorometer (Life Technologies, USA), optical density was determined using a NanoDrop (Thermo Fisher Scientific, MA, USA), and RNA integrity was assessed on an Agilent 2100 Bioanalyzer (Agilent, USA). Only the samples with an RNA integrity number (RIN) of more than 8.0 were selected for the preparation of small RNA libraries. Small RNA high-throughput sequencing was conducted with BGISEQ-500 (Beijing Genomics Institute, Shenzhen, China).

### Bioinformatic analysis of vsiRNAs and prediction of target genes

Small RNAs with lengths of 18–30 nt were screened for bioinformatic analysis after the preprocessing of raw data. To identify BYDV-GAV-derived siRNAs, clean reads were submitted to map the BYDV-GAV genome (accession number AY220739) using Bowtie software 2.0 permitting two mismatches [40]. The results were compared to those obtained with the miRNA database and Rfam. The sequences of clean reads that were identical or complementary to BYDV-GAV genomic sequences with zero mismatches and that could not be compared to the miRNA database and Rfam were considered query vsiRNAs and were chosen for target gene prediction by TargetFinder with default parameters [41]. Subsequently, GO and KEGG analyses were performed to analyze the biological functions of the target genes.

### RT-qPCR validation for target transcripts

The samples used for the RT-qPCR analysis of targets were inoculated with viruliferous aphids carrying BYDV-GAV at the three- to four-leaf stage, with 5 aphids being applied to each leaf, and nonviruliferous aphid-inoculated plants served as controls. Total RNA was extracted using the Biospin Plant Total RNA extraction kit (Bioer Technology, Hangzhou, China) according to the manufacturer's instructions. Next, 2 µg RNA was employed to perform reverse transcription with M-MLV reverse transcriptase (Promega, USA). For RT-qPCR, quantitative assays were conducted with an UltraSYBR Mixture (Cwbio, China) and a Roche LightCycler 480 Real-time PCR Detection System instrument (Roche, Switzerland). The gene-specific primer pairs of targets used for RT-qPCR were designed using Primer3web version 4.0.0 (https://primer3plus.com/primer3web/primer3web_input.htm), and the *TaEF* wheat gene was employed as the reference gene. All primers used in this study are listed in Additional file 1: Table S1. The RT-qPCR reaction system consisted of 12.5 μl of 2 × Ultra SYBR Mixture, 0.5 μl of forward primer, 0.5 μl of reverse primer, 1 μl of template DNA, and 10.5 μl of ddH_2_O with the following reaction conditions: 95℃ for 10 min followed by 35–40 cycles of 95℃ for 15 s and 60 ℃ for 1 min. The relative expression levels of targets were normalized using the 2^−△△Ct^ method [42]. All RT-qPCR reactions were repeated in three biological replicates and three technical replicates, and the means and corresponding standard errors were calculated.

### Virus-induced gene silencing (VIGS) vector construction and rub inoculation

Transient silencing of predicted target genes was implemented through virus-induced gene silencing (VIGS) with the BSMV vector. First, 200–300-bp fragments were amplified from Xiaoyan 6 wheat plants by PCR using Primer Star Max (Takara, Japan), and the primers were designed by Primer 5.0. All primers are listed in Additional file 1: Table S2. The three-component vectors of the BSMV were utilized for VIGS as described in a previous paper [43]. Next, the fragments of candidate genes were inserted into the γ components of BSMV using the One Step Cloning Kit (Vazyme, Nanjing, China), and empty vector-inoculated plants were utilized as a negative inoculation control, while γ-PDS (phytoene desaturase)-inoculated plants were employed as a positive inoculation control, and FES buffer-inoculated plants served as a negative control.

Subsequently, the three components of the BSMV were prepared by in vitro transcription using the RiboMAXTM Large Scale RNA Production Systems-T7 kit (Promega, America). BSMV α, β, and γ RNA transcripts were mixed at a ratio of 1:1:1 and added to FES buffer. Next, the mixtures were applied on the second leaves of the seedlings through rub inoculation, and three independent plants were employed for each treatment [44]. The infiltrated leaves were lightly sprinkled with water and kept in a growth chamber (25℃ for 24 h in the dark with high humidity, then under a 16-h light and 8-h dark photoperiod at a relative humidity of 75%).

### Estimation of gene silencing and BYDV-GAV content determination

Successfully infected plants were selected based on systemic mosaic symptoms of BSMV, and RT-qPCR was performed to determine the silencing efficiency of target genes at 10 d after inoculation [45]. The silenced plants and empty vector-inoculated plants were subsequently inoculated with viruliferous aphids for 7 d. The inoculated plants were collected, and RT-qPCR was performed to determine the virus contents of gene-silenced plants and control plants, while the *TaEF* gene was employed to normalize the data.

### Measurement of chlorophyll content

For each sample, approximately 200 mg of wheat leaf was collected from three independent replicate plants and cut into 1-mm-wide filaments. DMSO (2 mL) was added to the tubes, which were subsequently placed in a 65 °C incubator in the dark. The measurement of chlorophyll content was conducted after adding 8 mL 80% (v/v) acetone, and the absorbances at 663.6 and 646.6 nm were employed to determine the final quantitative value. Chlorophyll content can be calculated with the following formulas: Ca (mg∙L^−1^) = 12.27A663.6–2.52A646.6; Cb (mg∙L^−1^) = 20.10A646.6–4.92A663.6; CT = Ca + Cb = 7.35A663.6 + 17.58A646.6 [46].

### Cloning of the vsiRNA8856 and TaChlG genes

To determine whether the vsiRNA was derived from the target transcript, vsiRNA8856 was cloned using RT-PCR. First, small RNA was extracted from the BYDV-GAV-infected plants at 7 dpi using RNAiso reagent (TaKaRa Biotechnology Dalian Co., Ltd.), and second, cDNA was synthesized using TranScript miRNA First-Strand cDNA Synthesis SuperMix (Transgen Biotech, Beijing, China) according to the manufacturer’s instructions. Finally, PCR was performed by PrimerSTAR Max DNA Polymerase (TaKaRa Biotechnology Dalian Co., Ltd.). The vsiRNA-specific primers were employed as the forward primer, and the universal primer was utilized as the reverse primer. vsiRNAs were purified and cloned into the pMD19-T vector (TaKaRa Biotechnology Dalian Co., Ltd.) for sequencing. The primers employed for RT-PCR are listed in Additional file 1: Table S3.

The full-length sequence of *TaChlG* (TraesCS1D01G226100.1) was amplified by RT-PCR using PrimerSTAR Max DNA Polymerase (TaKaRa Biotechnology Dalian Co., Ltd.). Next, the product of gel extraction was constructed into the pMD19-T vector (TaKaRa Biotechnology Dalian Co., Ltd.) for sequencing. Primers used for RT-PCR are listed in Additional file 1: Table S3. The theoretic pI (isoelectric point) and Mw (molecular weight) of *TaChlG* were generated using the Compute pI/Mw tool online (https://web.expasy.org/compute_pi/). The prediction of subcellular localization was performed using the CELLO v2.5 web server [47].

### Multiple alignments and phylogenetic analysis

Multiple sequence alignments were performed using the ClustalW tool [48]. Next, a neighbor-joining (NJ) tree was generated by MEGA X software for phylogenetic analysis [49]. The bootstrap test method was set to 1000 replications.

### Target validation by 5′ RLM-RACE

5′ RLM-RACE (RNA ligase mediated rapid amplification of cDNA ends) assays were performed to validate the predicted targets. The samples of virus-infected wheat plants were harvested at 7 dpi, and total RNA was extracted using RNAiso Plus (Takara, China) according to the manufacturer’s instructions. The RACE assay was conducted with the FirstChoice™ RLM-RACE Kit (Invitrogen, US). The RNA was ligated with the 5′ RACE adaptor (GCUGAUGGCGAUGAAUGAACACUGCGUUUGCUGGCUUUGAUGAAA) using T4 RNA ligase. The ligated RNA was reverse-transcribed using random decamers and M-MLV reverse transcriptase. The cDNA was employed for nested PCR with two rounds of amplification with primers containing the 5′ RACE outer and inner primers supplied with the RLM-RACE kit and target gene-specific outer and inner primers designed by Primer 3. The first-round products served as the template to amplify the second round. Gel analysis of the products was conducted, and the expected bands were cloned into the pMD19-T vector for sequencing. The primers employed for this experiment are provided in Additional file 1: Table S4.

## Results

### Characteristics of vsiRNAs

**Fig. 2:**
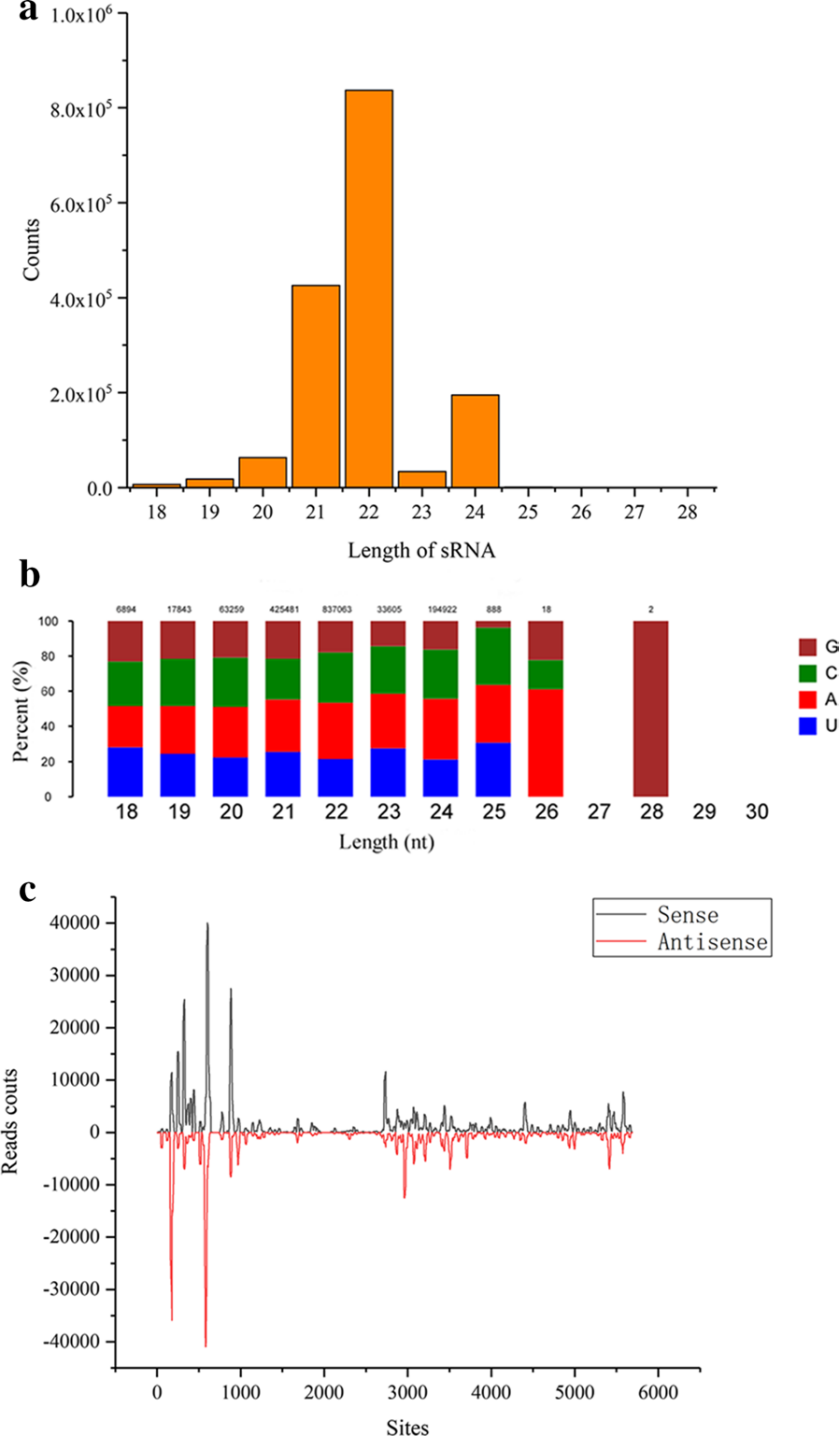
Characteristics of sRNAs. **a** Length distribution of sRNAs. The X-axis indicates the length of sRNA; the Y-axis indicates the number of total sRNA reads of different lengths in the BYDV-GAV-infected wheat plant through high-throughput sequencing. **b** The first nucleotide bias of vsiRNAs. The relative percentages of the four 5′-terminal nucleotides of the 18–30 nt vsiRNAs are shown in different colors. The X-axis indicates the sequence length, while the Y-axis shows the relative percentage of different nucleotides. **c** The polarity distribution of vsiRNAs in the positive and negative strands of the viral genome. The X-axis indicates the full-length of the BYDV-GAV genome, while the Y-axis shows the read counts. Reads mapped to the positive strand of the BYDV-GAV genome are shown in black, while reads mapped to the negative strand are shown in red

To determine the roles of vsiRNAs in the interactions between the virus and the host plant, high-throughput sequencing of small RNAs was performed on BGISEQ-500 to analyze the vsiRNA population in BYDV-GAV-infected Xiaoyan 6 wheat plants. A total of 26,286,625 clean reads were yielded from the library for further analysis. Next, we mapped the clean reads to the viral dsRNA genome with 2 mismatches and obtained 2,440,544 filtered reads, which accounted for 9.28%. After processing and redundancy screening, 113,884 filtered siRNAs were obtained, which were recognized as vsiRNAs. The length distribution of vsiRNAs indicated that 21- and 22-nt vsiRNAs were abundantly present followed by 24-nt vsiRNAs in the library (Fig. 2a). The results showed that BYDV-GAV infection produced a significantly increased population of 21- and 22-nt siRNAs, suggesting that DCL4 and DCL2 enzymes play major roles in the biogenesis of BYDV-GAV-derived siRNAs.

Previous studies reported that the specific recruitment of sRNAs by distinct AGO proteins largely depends on the 5′-terminal nucleotide [50, 51]. AGO2 and AGO4 primarily recruit small RNAs with a 5′-terminal A, whereas 5′-termini with U and C are preferentially recruited by AGO1 [52]. To investigate which AGO is primarily involved in the processing of BYDV-GAV-derived vsiRNAs, the 5′-terminal nucleotides of the vsiRNAs were analyzed. For the heavily accumulated 21- and 24-nt lengths, A and C accounted for a dominant proportion, while U and G were less abundant. According to the results, the BYDV-GAV-derived vsiRNAs had a 5′-terminal nucleotide bias toward A and U, which might be preferentially recruited by wheat AGO1, AGO2 and AGO4 to form the RISC (Fig. 2b).

To elucidate the origin of the BYDV-GAV-derived vsiRNAs, the polarity distribution of vsiRNAs in the positive ( +) and negative (-) strands of the viral dsRNA genome was characterized by aligning the vsiRNAs with the genome. The results indicated that the hotspots of sense- and antisense-vsiRNAs that were derived from positive-sense ( +)- and negative-sense ( −)-strands of BYDV-GAV genomic RNA were nearly the same (Fig. 2c). However, the number of sense-vsiRNAs was slightly greater than that of antisense-vsiRNAs.

From the distribution of vsiRNA in positive-sense ( +)- and negative-sense ( −)-strands of BYDV-GAV genomic RNA, we observed that vsiRNAs were not continuously distributed through the genomic segments. Among all segments, vsiRNAs predominantly accumulated in ORF1, OPF3, and ORF4, which were primarily responsible for viral replication, infection, and movement, suggesting that vsiRNAs play a vital role in the processing of viral invasion.

### Prediction of putative target transcripts of vsiRNAs

Target identification is beneficial to provide an in-depth understanding of the functions of vsiRNAs. In this study, vsiRNAs were employed to predict putative wheat target genes by the target analysis server TargetFinder with default parameters. A total of 23,719 target transcripts of 11,384 vsiRNAs were acquired after deleting duplicate items, of which 4,324 vsiRNAs had corresponding targets in wheat plants (Additional file 2: Table S5). Annotation of the predicted target genes was performed referring to the wheat transcript database from ensemble plants.

### GO and KEGG pathway enrichment analysis of predicted targets

**Fig. 3:**
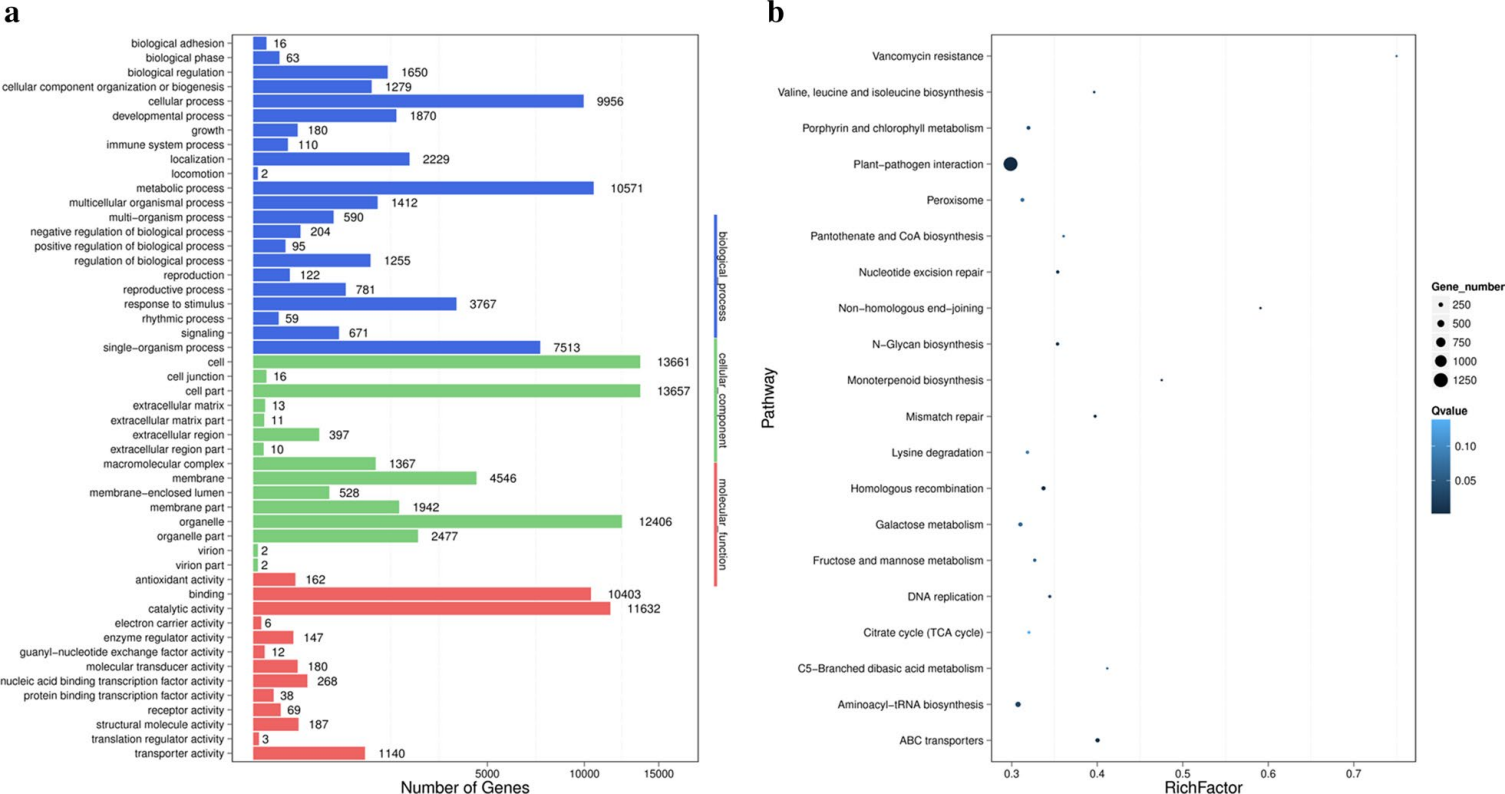
Gene ontology (GO) and KEGG analysis of predicted target genes in wheat. **a** GO enrichment analysis of the predicted target genes. The X-axis represents the number of genes, and the Y-axis represents the functional category. The numbers indicate the numbers of the genes related to corresponding functions. **b** KEGG enrichment analysis of the predicted target genes. The statistics of the potential target gene path enrichment of the vsiRNAs were conducted. The X-axis indicates the ratio of the numbers of target genes located in the pathway items, while the Y-axis represents the functional category

To determine the roles of the predicted target genes in wheat plants, GO and KEGG pathway enrichment analyses were performed to investigate the underlying functions. The GO enrichment analysis demonstrated that cellular and metabolic processes were the most significantly represented groups in the biological process category, cells, and cell parts were the most commonly enriched terms in the cellular component category, while catalytic activity and binding were the predominantly enriched groups within the molecular function category (Fig. 3a). The KEGG pathway enrichment analysis showed that the target genes were most abundant in enriched plant-pathogen interaction pathways (Fig. 3b). From the KEGG pathway, we also observed that the porphyrin and chlorophyll metabolism pathway was enriched, containing approximately 178 genes. We speculate that this pathway was involved in the yellowing of BYDV-GAV-infected wheat.

### Expression of genes related to yellowing of BYDV-GAV-infected wheat

**Fig. 4:**
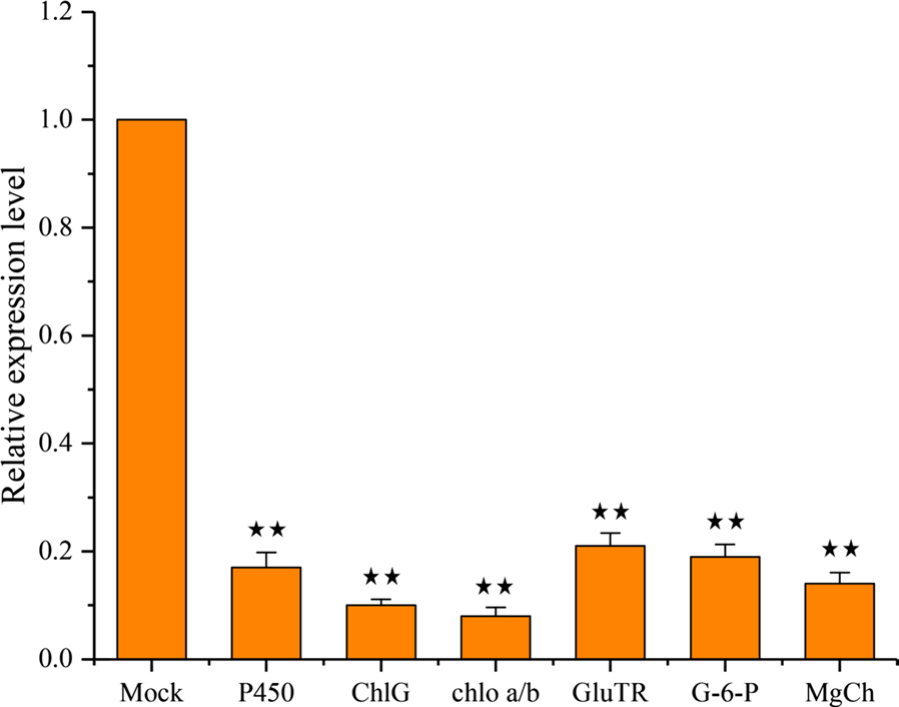
Validation of the relative expression levels of selected targets by RT-qPCR after BYDV-GAV infection. P450: cytochrome P450; ChlG: chlorophyll synthase; chlo a/b: chlorophyll a-b binding protein; GluTR: glutamyl-tRNA reductase; G-6-P: glucose-6-phosphate isomerase; MgCh: chloroplast Mg-chelatase subunit. The expression of transcripts was normalized by the level of *TaEF* in RT-qPCR. Three biological replicates were performed for each target. Error bars represent standard errors of replicates. Statistical differences were assessed using Student’s t-tests. Double asterisks indicate a significant difference at P < 0.01

To identify the genes related to symptoms of yellowing, we selected a series of predicted targets involved in chlorophyll biosynthesis. Next, RT-qPCR was conducted to measure their relative expression level in wheat samples at 14 d after infection with BYDV-GAV, and we found that most of the targets showed a significantly decreased expression level. For example, the relative accumulation levels of cytochrome P450, chlorophyll synthase, chlorophyll a-b binding protein, glutamyl-tRNA reductase, glucose-6-phosphate isomerase, and chloroplast Mg-chelatase subunit were decreased by approximately 0.17-, 0.1-, 0.08-, 0.21-, 0.19-, and 0.14-fold, respectively (Fig. 4). The results indicated that infection with BYDV-GAV can inhibit the expression of some chlorophyll biosynthesis-related genes in wheat plants. Therefore, further study is urgently needed to confirm whether vsiRNAs had a downregulation effect on these predicted target genes. All of these predicted target mRNA cleavage sites of the vsiRNAs are listed in Additional file 3 Table S6.

### ChlG-silenced wheat displays leaf yellowing

**Fig. 5:**
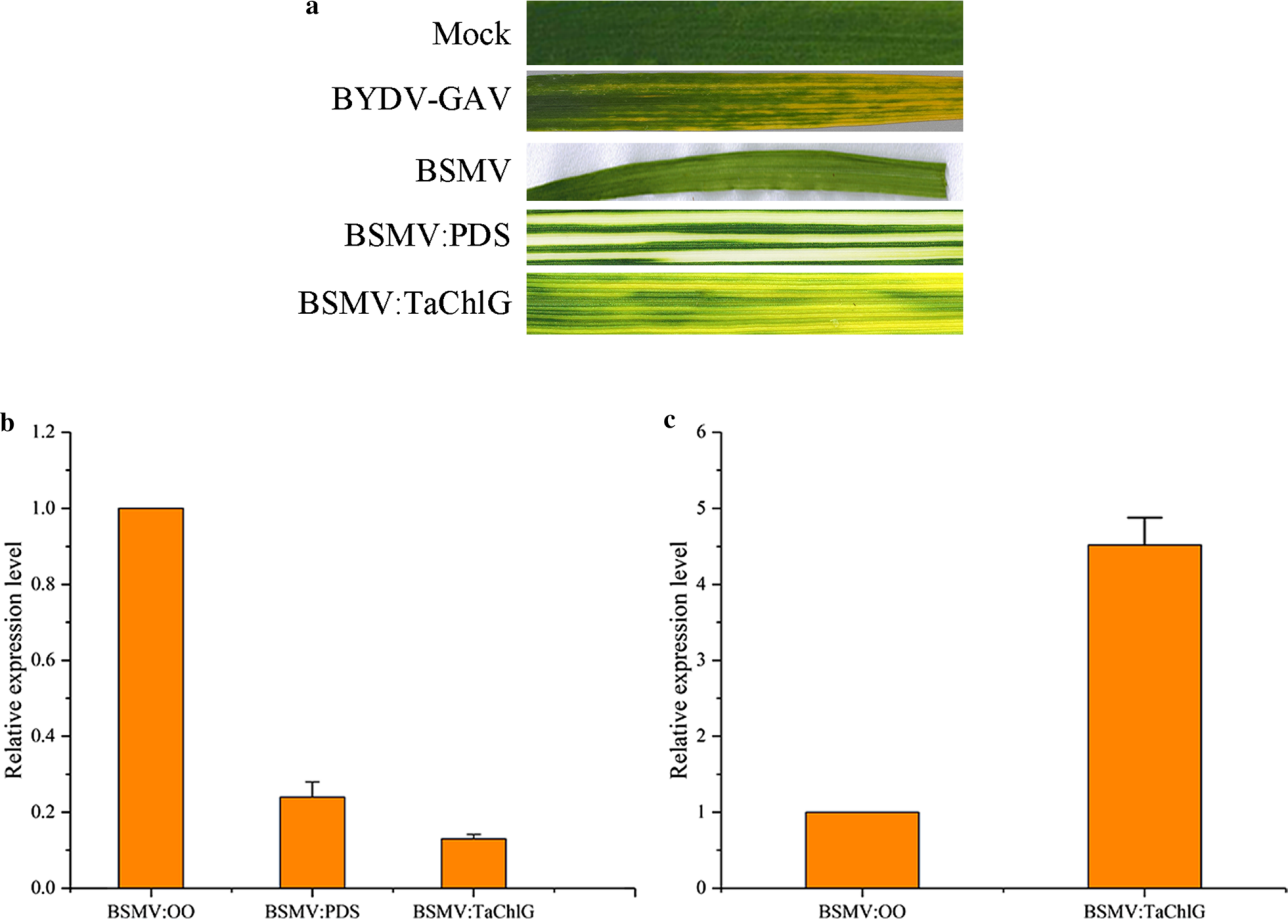
Silencing of TaChlG by BSMV. **a** The phenotype in the fourth leaves of healthy plants, BYDV-GAV inoculated plants, BSMV empty vector inoculated plants, PDS-silenced plants, and TaChlG-silenced plants. Photographs were taken at 10 d postinoculation (dpi). **b** Relative expression levels of PDS and TaChlG in PDS-silenced and TaChlG-silenced plants, respectively. The BSMV empty vector inoculated plants were used as the control. Each relative expression level is presented from three biological samples, and each biological sample had three technical replicates. Error bars represent standard errors of replicates **c** Relative expression levels of BYDV-GAV in TaChlG-silenced plants. The CP of BYDV-GAV was used to determine the accumulation of virus. *TaEF* acted as an internal control for normalization in RT-qPCR

Among the targets, ChlG was predicted to encode chlorophyll synthase, which functions in the last step of the chlorophyll biosynthetic pathway, playing a decisive role in chlorophyll content. To determine the functions of the target gene, transient silencing of ChlG was implemented through virus-induced gene silencing (VIGS) with the BSMV system. At 10 d post-infiltration (dpi), we determined that the plants infiltrated by γ-PDS exhibited significant photobleaching, whereas the newly emerged leaves of ChlG-silenced wheat plants were yellowing, which is the typical symptom of BYDV-GAV (Fig. 5a). Meanwhile, the expression levels of ChlG and PDS genes in silenced plants were detected by RT-qPCR. The results showed that the expression level of the PDS gene was decreased by almost 75% and that the ChlG gene was reduced by 87% when compared to the negative control, which was inoculated with the empty vector (Fig. 5b).

After 7 d of infection with viruliferous aphids carrying BYDV-GAV, total RNA of the inoculated leaves was extracted to analyze the relative content of the virus. The CP gene of BYDV-GAV was employed to determine viral accumulation. The RT-qPCR results demonstrated that the accumulation levels of BYDV-GAV in ChlG-silenced wheat plants showed a greater than 4.5-fold increase compared to the negative control plants (Fig. 5c). The increased viral accumulation in ChlG-silenced wheat plants indicated that ChlG might be involved in the resistance to BYDV-GAV infection.

### Alteration of chlorophyll content in wheat and ChlG-silenced wheat plants

**Fig. 6:**
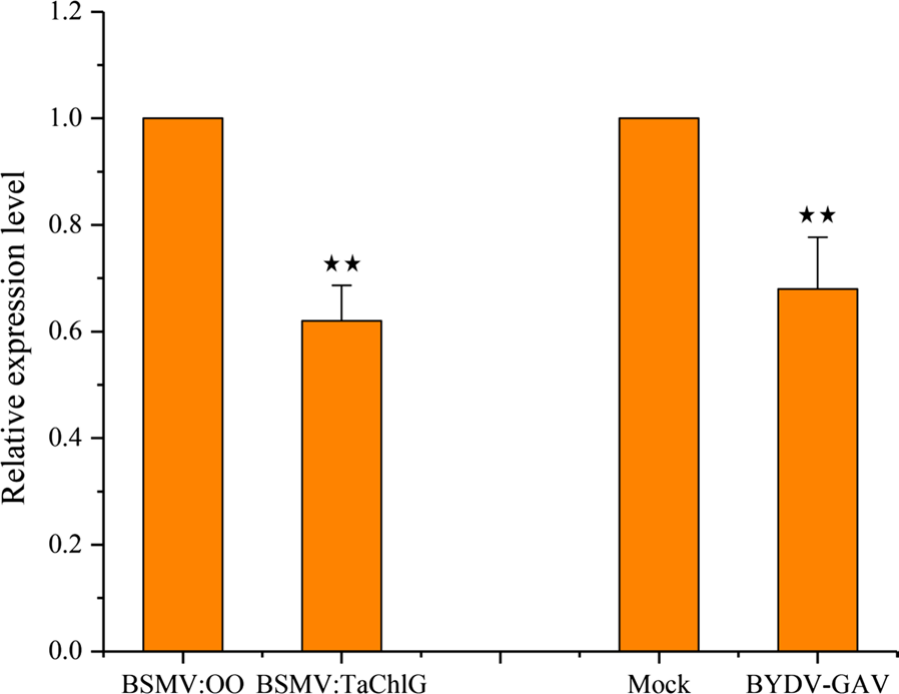
Chlorophyll content in TaChlG-silenced wheat plants. The determination of chlorophyll was conducted at 10 dpi after taking pictures. Each relative expression level contained three biological samples, and each biological sample had three technical replicates. Statistical differences were assessed using Student’s t-tests. Double asterisks indicate a significant difference at P < 0.01

To further confirm the relationship between chlorophyll content and the expression of the ChlG gene in wheat plants, we measured the chlorophyll content by utilizing the absorbance at 663.6 and 646.6 nm to calculate the final quantitative value. Both wheat and ChlG-silenced wheat plants infected with viruliferous aphids were collected to analyze the chlorophyll content. The chlorophyll content in BYDV-GAV inoculated wheat plants showed a significant decrease of approximately 32% compared with mock-inoculated plants at 10 dpi. As expected, this accumulation was also significantly reduced by 38% in the ChlG-silenced wheat plants compared to the mock-inoculated plants at 10 dpi (Fig. 6). The results indicated that the yellowing symptom of BYDV-GAV might be closely associated with the destruction of ChlG.

### vsiRNA8856 from the common region of MP and CP

**Fig. 7:**
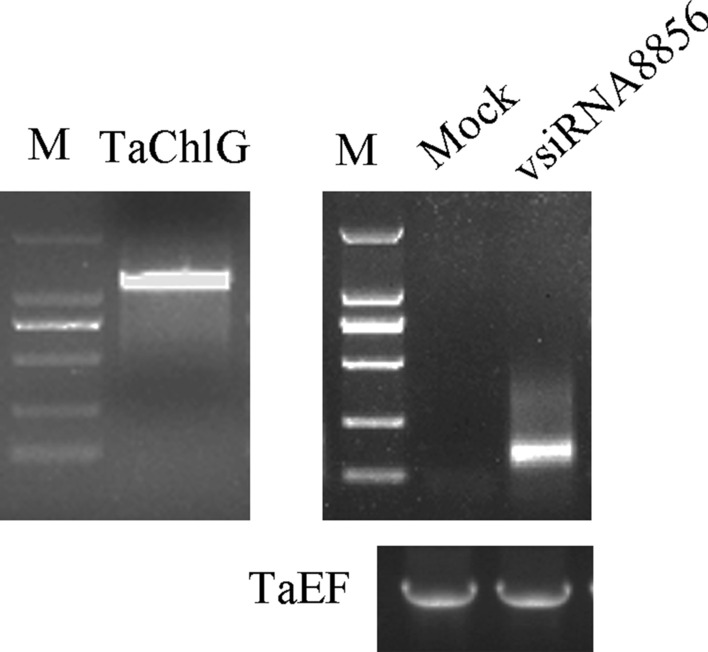
Amplification of TaChlG and vsiRNA8856 by RT-PCR. Total RNA was extracted from plants inoculated with BYDV-GAV. *TaEF* was used as an internal control. Gel analysis of products was conducted, and expected bands were cloned into the pMD19-T vector for sequencing

Previous studies have reported that vsiRNAs can target host endogenous gene transcripts in plants [20, 21, 27]. To explore the relationship between the decreased expression of ChlG and the BYDV-GAV-derived vsiRNAs, we analyzed the results of the vsiRNA that was predicted to target ChlG. We found the cleavage site primarily at the 30-, 75-, and 1097-bp sites of the ChlG transcript. Then, the expression of vsiRNAs was detected in wheat plants by RT-PCR (Fig. 7). In addition, we found the production of large amounts of vsiRNA8856 along with BYDV-GAV infection, and 346 reads were detected from the sequencing data.

### Validation of vsiRNA target by 5′ RLM-RACE

**Fig. 8:**
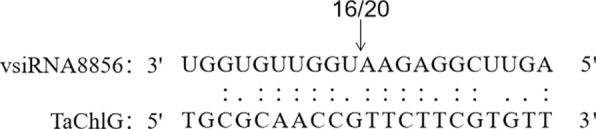
Validation of target genes by 5′ RLM-RACE. The confirmed cleavage sites are indicated by vertical arrows with the frequency of cleavage

Although our results show that downregulation of ChlG correlated strongly with symptoms of BYDV-GAV, it is not known how viral infection downregulates host genes. To identify the accuracy of predicted vsiRNA targets, 5′ RLM-RACE was used to confirm the interaction between vsiRNAs and predicted target genes. Then, cleavage sites were found between the 10th and 11th nucleotides of vsiRNA8856 for the ChlG transcript. The results of 5′ RLM-RACE further indicate that the vsiRNAs cleave their corresponding target genes at specific sites (Fig. 8), which suggested that the downregulation of ChlG contributed to cleavage by vsiRNAs from BYDV-GAV.

### Cloning, multiple alignments and phylogenetic analysis of ChlGs

The full-length ChlG gene was cloned from wheat plants by RT-PCR. The results showed that the sequence of the ChlG gene is 1134 bp with eight ORFs, of which ORF1 contains the complete sequence. The ChlG protein contains a total of 377 amino acids (aa) with a molecular weight of 40.6 kDa and a theoretical pI of 7.59. The subcellular localization prediction suggests that the ChlG gene may be located at the plasma membrane.

**Fig. 9:**
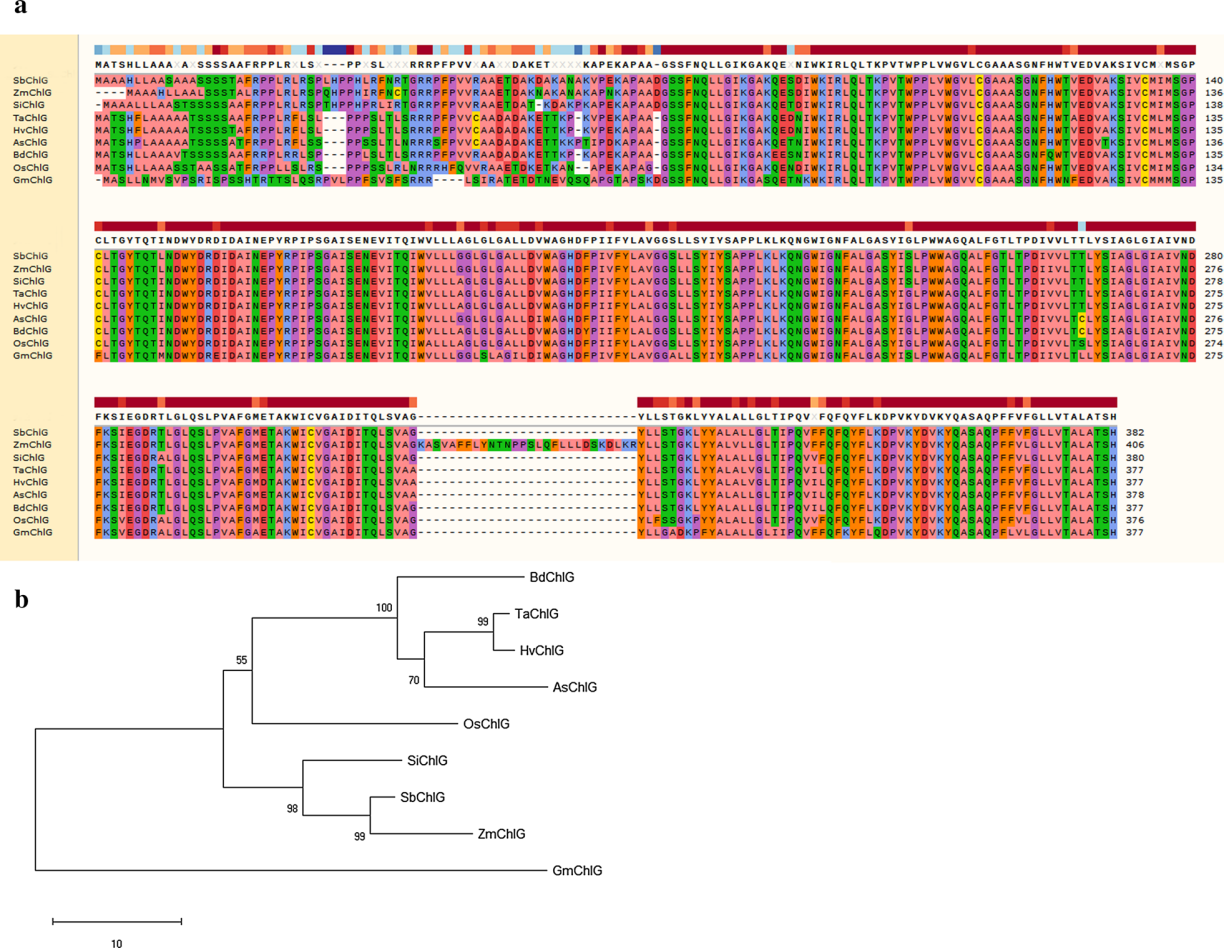
Multiple alignments and phylogenetic analysis using ChlG aa sequences from different plant species. **a** Multiple sequence alignment used the Triticum aestivum ChlG sequence and its closely related ChlG protein sequences from other plant species. The ChlG in these plant species is presented as *Hordeum vulgare* (HvChlG), *Oryza sativa* (OsChlG), *Brachypodium distachyon* (BdChlG), *Sorghum bicolor* (SbChlG), *Setaria italica* (SiChlG), *Zea mays* (ZmChlG), *Avena Sativa* (AsChlG), *Glycine max* (GmChlG) and *Triticum aestivum* (TaChlG). The amino acids with identity greater than 50% are labeled with colored boxes. **b** A phylogenetic tree was constructed with the results of sequence alignment. The phylogenetic tree was constructed using the NJ (neighbor-joining) method with 1000 bootstrap replications

To further evaluate the phylogenetic relationships between wheat ChlG and other homologous species, we aligned the aa sequences among the species *Hordeum vulgare* (HvChlG), *Oryza sativa* (OsChlG), *Brachypodium distachyon* (BdChlG), *Sorghum bicolor* (SbChlG), *Setaria italica* (SiChlG), *Zea mays* (ZmChlG), *Avena sativa* (AsChlG), *Glycine max* (GmChlG) and *Triticum aestivum* (TaChlG) (Fig. 9a). The sequence alignment results showed that ChlG shares high aa sequence identities. In addition, we constructed a phylogenetic tree using these aa sequences (Fig. 9b). The results indicated that *TaChlG* is most closely related to *HvChlG* followed by *BdChlG* and *AsChlG*. It has been indicted that BYDV-GAV can infect *Hordeum vulgare*, *Avena sativa*, and *Brachypodium distachyon* and cause symptoms. Hence, we infer that ChlGs have similar functions that were targeted by BYDV-GAV for the production of symptoms in these closely related species.

## Discussion

In eukaryotes, RNA silencing acts as a conserved posttranscriptional regulation mechanism of gene expression that widely occurs in antiviral silencing. Viral infection is accompanied by the production of a large number of siRNAs derived from the viral genome in infected plant cells, triggering widespread silencing of host genes. In this study, we performed a high-throughput small RNA sequencing approach to profile vsiRNA populations from BYDV-GAV inoculated wheat plants and to provide experimental evidence supporting the association of chlorophyll biosynthesis with leaf yellowing symptoms.

Statistical analysis of small RNA sequencing data demonstrated that BYDV-GAV infection resulted in the production of large amounts of vsiRNAs. DCLs, AGOs, and RNA-dependent RNA polymerase (RDR) proteins are core components of plant RNA silencing pathways for the production and amplification of viral-derived siRNAs [53, 54]. Previous studies showed that DCL2 and DCL4 process invasive viral RNAs into 22- and 21-nt siRNAs, respectively, in *Arabidopsis* [55]. Among these vsiRNAs, DCL4-dependent 21-nt vsiRNAs play a dominant role in positive-sense RNA virus-infected plants, whereas DCL2-dependent 22-nt vsiRNAs can substitute for DCL4 when the expression of DCL4 is inhibited [53, 56]. vsiRNA profiling of Southern rice black-streaked dwarf virus (SRBSDV)-infected rice plants showed that 21- and 22-nt were the most abundant vsiRNAs, suggesting that DCL4 and DCL2 are the primary RNA silencing components for generating vsiRNAs [57]. The vsiRNAs obtained from rice black-streaked dwarf virus (RBSDV)-infected maize and rice plants accumulated preferentially as 21- and 22-nt molecules [23, 58]. In conclusion, the different length distributions of vsiRNAs might represent the difference between the biosynthetic pathways of vsiRNAs. In this study, we found that 21- and 22-nt vsiRNAs derived from BYDV-GAV-infected wheat plants accumulated at high levels, suggesting that DCL4 and DCL2 play a dominant role in the production of vsiRNAs (Fig. 1).

The 5′-terminal nucleotide of sRNAs corresponds to different AGO complexes. For example, sRNAs with a 5′-terminal A are preferentially recruited by AGO2 and AGO4, whereas sRNAs with a 5′-terminal U favor AGO1 in *Arabidopsis thaliana* [50]. These findings show that the role of AGO1, AGO2, and AGO4 is consistent with the performance of sRNAs, which have a strong 5′-terminal A/U base preference [59]. In BYDV-GAV-infected wheat plants, the emergence of 5′-terminal A was the most frequent in the 21- and 22-nt vsiRNAs, suggesting that the vsiRNAs would be primarily loaded into AGO2 and AGO4 (Fig. 1).

Virus infection is often accompanied by the production of a large number of vsiRNAs, causing changes in the expression of host transcripts, for example, RSV- and CMV-infected *N. benthamiana* plants, CWMV-infected wheat plants, sugarcane mosaic virus (SCMV)-inoculated maize plants and cucumber green mottle mosaic virus (CGMMV)-inoculated cucumber plants [20, 24, 27, 60, 61]. Increasing evidence suggests that vsiRNAs can silence specific host mRNAs when there is a near-perfect complementary, which plays an important role at the posttranscriptional level [20, 24]. These findings indicate that vsiRNA-mediated host gene silencing contributes to the development of disease symptoms caused by a viral infection. However, there is still a lack of detailed experimental evidence regarding the interaction between BYDV-GAV vsiRNAs and host target transcripts. In this study, small RNA sequencing was conducted to profile BYDV-GAV-derived vsiRNA, and psRobot and TargetFinder software were used to predict vsiRNA targets. To elucidate the roles of vsiRNAs in the production of leaf yellowing in BYDV-GAV-inoculated wheat plants, we selected a variety of chlorophyll biosynthesis- and chloroplast-related genes based on leaf yellowing symptoms to perform further analysis. The quantitative expression analysis of those selected targets indicated that most of the predicted vsiRNA targets were downregulated. This result suggests that virus-induced posttranscriptional gene silencing mechanisms play a major role in regulating BYDV-GAV-infected wheat plants.

Plant chlorophyll biosynthesis is composed of multiple complex enzymatic steps, including the synthesis of aminolevulinic acid (ALA), assembly of ring-structure, Mg chelation, methyltransferase, catalyzes the formation of chlorophyllide and chlorophyll synthase [38, 62]. Chlorophyll synthase (CHLG) is located at the last step of the chlorophyll biosynthetic pathway. The production of chlorophyll is accomplished by chlorophyll synthase (CHLG) esterifying chlorides a and b. Hence, the chlorophyll content is, to a great extent, determined by the normal expression of CHLG. Transgenic tobacco plants expressing CHLG enhanced ALA synthesizing capacity and increased chelatase activities, whereas the transcript levels of chlorophyll biosynthesis genes and chlorophyll-binding proteins were downregulated in response to reduced CHLG expression [38].

Infection with plant viruses is often associated with plant symptoms [21, 24, 63]. A previous study indicated that BYDV-GAV infection was associated with the downregulated expression of chlorophyll biosynthesis and chloroplast-related transcripts, which may be the source of wheat leaf yellowing [39]. The vsiRNA-mediated virus-host interaction mechanism reported here is consistent with previous observations. In our study, we identified a chlorophyll synthetase gene that was targeted by vsiRNA8856, which is associated with the production of wheat leaf yellowing symptoms. It is well known that chlorophyll biosynthesis-related enzymes are proposed to play crucial roles in chlorophyll biosynthesis and the alteration of leaf color [34, 64]. We used RT-qPCR to analyze the change in the content of CHLG and found that infection with BYDV-GAV suppressed its expression. Virus-induced gene silencing (VIGS) is an effective tool to study plant endogenous gene function and has been used extensively in wheat [43]. From the results of RT-qPCR in TaChlG-silenced plants, the relative expression level of BYDV-GAV was significantly increased. In addition, the yellowing symptoms of CHLG-silenced wheat plants were consistent with the symptoms of BYDV-GAV infection, suggesting that CHLG might be the target of viral invasion.

5′ RACE has been used to identify sRNA-mediated cleavage sites [65]. From the 5′ RACE results, vsiRNA8856 specifically cleaves CHLG mRNA at the 10th-11th nucleotide. This finding is similar to the result that vsiRNAs from SCMV-inoculated maize plants targeted host transcripts for cleavage [60]. We also found that vsiRNA8856 comes from the overlap region of MP and CP, which is responsible for viral systemic movement and replication, respectively, in wheat plants [66]. Additionally, the results of target prediction in this study indicated that some other vsiRNAs might target the chlorophyll biosynthesis pathway, and chloroplast-related genes, for example, glucose-6-phosphate isomerase, chloroplast Mg-chelatase subunit, glutamyl-tRNA reductase, cytochrome P450, and chlorophyll a/b-binding protein, served as the targets of multiple BYDV-GAV vsiRNAs, which are essential for the production of chlorophyll content [36, 67–69]. The quantitative analysis results of these genes were consistent with the speculation that the expression level of these genes showed a significant decrease after BYDV-GAV infection (Fig. 4). The disruption of these genes makes it a favorable opportunity for viruses to invade host plants and symptom formation.

BYDV-GAV can not only infect wheat but also infect other cereal crops, such as barley, *Zea mays*, *Brachypodium distachyon,* and oat [3, 70, 71]. The infection often causes viral symptoms. The results of multiple alignments and phylogenetic analysis revealed that there is some commonality in these host plants, perhaps the targets of BYDV-GAV-derived vsiRNAs. Therefore, our results can provide some insights for understanding the infection of BYDV-GAV by other species.

Our study performed small RNA sequencing, RT-qPCR, VIGS, and chlorophyll-level analysis to explore the interactions involved in the yellowing symptoms of leaves and vsiRNAs derived from BYDV-GAV in susceptible wheat plants. The results indicated that after BYDV-GAV infection, the decreased expression of chlorophyll biosynthesis and chloroplast-related genes may contribute to the lower chlorophyll content, consequently causing leaf yellowing. In summary, BYDV-GAV infection in susceptible wheat successfully causes the development of symptoms by producing a large amount of vsiRNAs to widely target host transcripts. Although our results indicate that BYDV-GAV-derived vsiRNAs play an important role in viral infection and symptom production, the molecular mechanisms underlying this phenomenon have not been elucidated. Further study needs to be carried out on the biological roles and functional mechanisms underlying specific BYDV-GAV-derived vsiRNAs in the future.

## Conclusions

In this study, we profiled vsiRNAs in BYDV-GAV-infected wheat plants and investigated their potential roles in targeting some wheat genes involved in leaf yellowing. We found a vsiRNA8856-targeted chlorophyll synthetase gene for specific cleavage. Our study provides experimental evidence that vsiRNAs derived from BYDV-GAV can target host genes for symptom development.

## Supplementary information

**Additional file 1**: **Table S1**. List of the RT-qPCR primers used for validation of target transcripts in this study. **Table S2**. List of the primers used for VIGS vector construction, detection of silencing efficiency, and determination of viral. **Table S3**. List of the RT-PCR primers used for cloning of vsiRNA8856 and the TaChlG gene. **Table S4**. List of the PCR primers used for 5’RLM-RACE.

**Additional file 2**: **Table S5**. The prediction results of putative target transcripts of vsiRNAs.

**Additional file 3**: **Table S6**. The results of predicted target mRNA cleavage sites of the vsiRNAs.

## Data Availability

All data supporting the conclusions of this article are included in this published article.

## Abbreviations

BYDV: Barley yellow dwarf virus
vsiRNAs: Virus-derived siRNAs
ORF: Open reading frame
AGO: Argonaute
DCL: Dicer-like
RDRP: RNA-dependent RNA polymerase
RISC: RNA-induced silencing complex
RdDM: RNA-directed DNA methylation
CHLG: Chlorophyll synthase
ChRGs: Chloroplast-related genes
GO: Gene Ontology
KEGG: Kyoto Encyclopaedia of Genes and Genomes
RT-qPCR: Reverse transcription-quantitative PCR
dpi: Days postinoculation
RACE: RNA ligase mediated rapid amplification of cDNA ends
